# LINE-1-Induced Retrotransposition Affects Early Preimplantation Embryo DNA Integrity and Pluripotency

**DOI:** 10.3390/ijms252312722

**Published:** 2024-11-27

**Authors:** Prodromos Sakaloglou, Leandros Lazaros, Ioanna Bouba, Sofia Markoula, Athanasios Zikopoulos, Eirini Drakaki, Ismini Anagnostaki, Anastasios Potiris, Sofoklis Stavros, Angeliki Gerede, Ekaterini Domali, Peter Drakakis, Theodoros Tzavaras, Ioannis Georgiou

**Affiliations:** 1 Laboratory of Medical Genetics and Human Reproduction, School of Health Sciences, Faculty of Medicine, University of Ioannina, 451 10 Ioannina, Greece; pr.sakaloglou@gmail.com (P.S.); leandroslazaros@yahoo.com (L.L.); ioannabouba@gmail.com (I.B.); smarkoula@grads.uoi.gr (S.M.); 2 Medical Genetics and Assisted Reproduction Unit, Department of Obstetrics and Gynecology, Ioannina University Hospital, 451 10 Ioannina, Greece; 3 Third Department of Obstetrics and Gynecology, University General Hospital “ATTIKON”, Medical School, National and Kapodistrian University of Athens, 124 62 Athens, Greece; thanzik92@gmail.com (A.Z.); isanagnostaki3@gmail.com (I.A.); apotiris@med.uoa.gr (A.P.); sfstavrou@med.uoa.gr (S.S.); pdrakakis@med.uoa.gr (P.D.); 4 First Department of Obstetrics and Gynecology, Alexandra Hospital, Medical School, National and Kapodistrian University of Athens, 115 28 Athens, Greece; eirinidrak@med.uoa.gr (E.D.); kdomali@yahoo.fr (E.D.); 5 Department of Obstetrics and Gynecology, Democritus University of Thrace, 691 00 Alexandroupolis, Greece; agerede@otenet.gr; 6 Department of General Biology, School of Health Sciences, Faculty of Medicine, University of Ioannina, 451 10 Ioannina, Greece; thtzavar@uoi.gr

**Keywords:** DNA methylation, double-strand DNA breaks, pluripotency factors, preimplantation embryo development, retroelements

## Abstract

Retrotransposable elements are implicated in genome rearrangements and gene expression alterations that result in various human disorders. In the current study, we sought to investigate the potential effects of long interspersed elements-1 (LINE-1) overexpression on the integrity and methylation of DNA and on the expression of three major pluripotency factors (OCT4, SOX2, NANOG) during the preimplantation stages of human embryo development. Human MI oocytes were matured in vitro to MII and transfected through intracytoplasmic sperm injection (ICSI) either with an EGFP vector carrying a cloned active human LINE-1 retroelement or with the same EGFP vector without insert as control. The occurrence of retrotransposition events was screened by fluorescent microscopy. The in vitro preimplantation development as well as the methylation, pluripotency, and DNA double-strand breaks (DSBs) of the transfected embryos were examined. LINE-1 retrotransposons gave rise to new retrotransposition events in the transfected embryos. LINE-1 injected embryos were characterized by accelerated asymmetrical cell division, multiple cellular fragments, cleavage arrest, and degeneration. Early OCT4 expression remained unaltered, but cleavage arrest and a high fragmentation rate hindered the expression of SOX2/NANOG at the morula stage. Increased DNA DSBs were observed in cleavage-stage blastomeres, while no methylation changes were detected before the cleavage arrest. Our data provide evidence that LINE-1 retrotransposition in human preimplantation embryos may induce DNA DSBs, while at the same time, it appears to interfere with the expression patterns of pluripotency factors. The morphological, structural, and cleavage abnormalities of the transfected embryos show that aberrant retroelement expression may negatively affect human embryo development.

## 1. Introduction

Mammalian preimplantation embryo development is characterized by DNA methylation reprogramming, pluripotency factors expression, and chromatin integrity alterations, which occur between the fertilization and the full-growth preimplantation blastocyst. The paternal genome undergoes active demethylation post-fertilization, whereas the maternal genome is passively demethylated from the first mitotic division to the morula stage [1,2]. However, imprinted genes reserve their methylation status during the massive demethylation process of the genome [3,4]. De novo methylation occurs at the early blastocyst stage, which coincides with the initiation of differentiation events in the preimplantation embryo, resulting in hypermethylated inner cell mass (ICM) and hypomethylation of the trophectoderm (TE). This asymmetry is maintained during development, leading to hypermethylated somatic tissues and undermethylated extraembryonic tissues, such as the placenta [2,5].

The pluripotency and self-renewal of embryonic stem (ES) cells are controlled by transcriptional networks, extra-cellular signaling, and epigenetic modifications, which activate or silence numerous genes that encode transcriptional factors and co-regulators [6,7,8]. OCT4, a transcriptional factor of the POU family, which promotes or suppresses the expression of target genes, regulates the pluripotency maintenance of ES cells and ICM formation [9]. OCT4 is initially expressed in 4–8-cell mouse embryos, presenting an expression peak in the blastomeres of morula stage embryos, whereas its expression in blastocyst stage embryos is limited to ICM [10]. SOX2 transcriptional factor, a member of the SOX family, binds to the DNA through the ‘high-mobility group’ region and regulates histone modifications and transcriptional events [11]. SOX2 expression is detected in various cells of morula stage mouse embryos, while in blastocyst stage embryos is expressed only in ICM cells. Finally, NANOG transcriptional factor presents the same expression pattern as SOX2 [6,12,13]. High NANOG levels promote the expression of other pluripotency factors, thereby maintaining ESC pluripotency, while low NANOG levels promote the differentiation of ES cells to the endoderm [14].

The above transcriptional factors interact with various epigenetic factors, such as polycomb repressive complexes (PRCs), in order to regulate the transcriptional activity of genes involved in cell differentiation [13,15]. ESC differentiation processes depend on gene expression variability, which requires a genome with high plasticity. Previous studies have shown that the double-stranded form of DNA and the organization of chromatin play crucial roles in genome plasticity. Chromatin, modifying the affinity of transcriptional factors with the binding sites, controls the access of regulatory factors to gene promoters. The presence of DNA double-strand breaks can compromise the above processes. Furthermore, the chromatin protein dynamics influence the degree of chromatin compaction, which in turn affects transcriptional factors’s access to the regulatory regions of the genes [16,17] and consequently, pluripotency conservation and ESC differentiation. Moreover, pluripotency factors interfere with cell cycle control check points through ATM, p53, and RB pathways [18,19].

Retrotrasposition has played a crucial role in human genome configuration during evolution. Retrotransposons are endogenous genetic elements comprising approximately 90% of all transposable elements and 45% of the human genome [20,21]. Long interspersed elements-1 (LINE-1, L1), which are autonomous non-long terminal repeat (non-LTR) retrotransposons with more than half a million copies, represent 17% of the human genome. LINE-1 retrotransposons encode proteins with RNA-binding, endonuclease, and reverse transcriptase activities [22,23]. LINE-1-based recombination and retrotransposition, apart from simple self-insertion, can also affect the human genome structure, causing DNA strand breaks [22], deletions [24], mutations [25], and genomic instability [26]. The research hypothesis related to whether double-strand DNA breaks occur due to retrotransposition of LINE-1 in preimplantation embryos, affecting further embryo development, DNA integrity, and pluripotency marker expression in the dividing blastomeres. In the current study, we explored the effects of retrotransposition and in particular, those of LINE-1 expression on DNA methylation reprogramming, expression of pluripotency factors (OCT4, SOX2, NANOG), and genome integrity during the preimplantation stages of human embryo development.

## 2. Results

**Figure 1:**
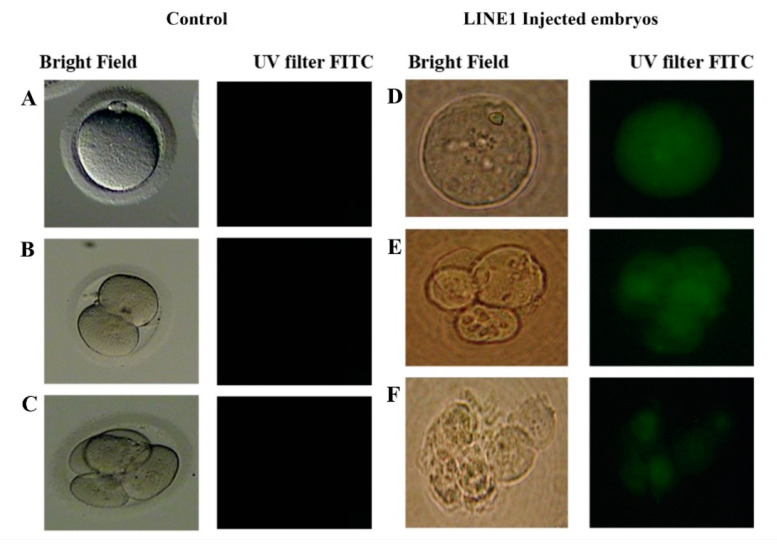
Active retrotransposition was observed in LINE1-injected embryos (**D**–**F**) and was absent in normal non- injected human preimplantation embryos (**A**–**C**). Retrotransposition of LINE 1 detected through EGFP expression (FITC filter, right column) with an Olympus fluorescence microscope: (**A**,**D**) fertilized oocytes, (**B**) 2-cell embryo, (**C**,**E**) 4-cell embryo, (**F**) 6-cell embryo with fragments.

**Figure 2:**
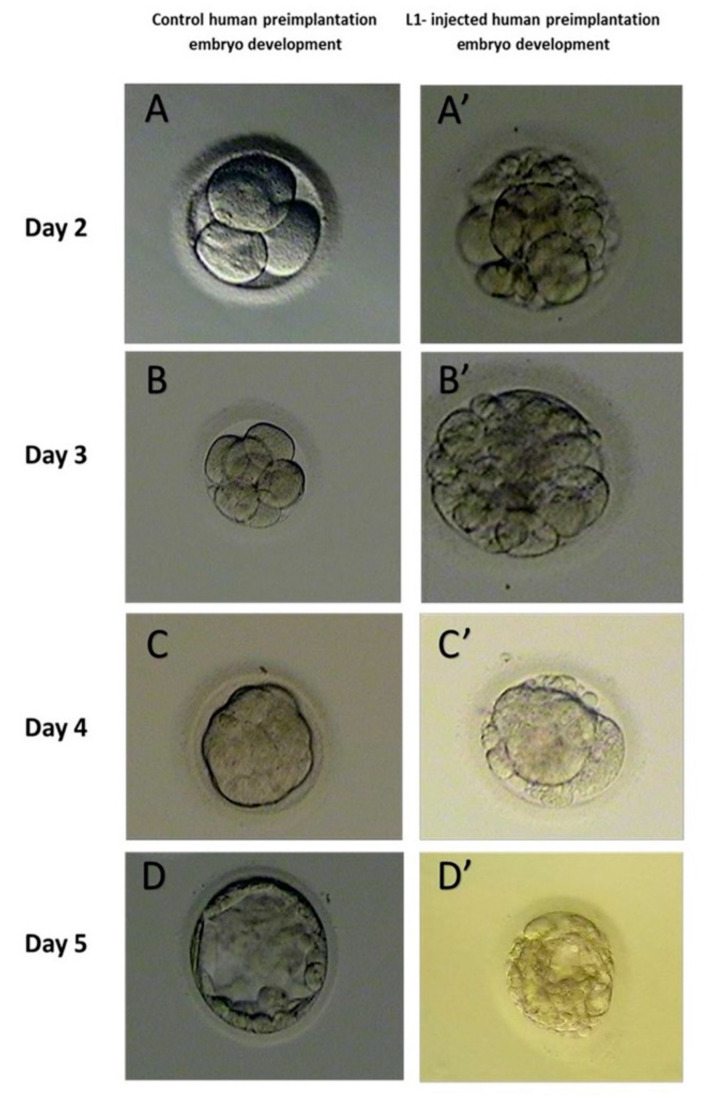
Impaired human preimplantation embryo development from control and LINE-1 injected oocytes donated for research. From Day 2 to Day 5, the development of injected and contaminated embryos by human LINE-1 was affected by excessive blastomere fragmentation, incomplete morula (fragmentation, incomplete, or lack of compaction of morula), abnormal blastocyst cavitation, and inner cell mass formation (**A’**–**D’**). (**A**–**D**) Normal embryos obtained from control oocytes at the same developmental stage as LINE-1 injected embryos.

Embryos at the cleavage stage are graded based on the appearance of the cells under a light microscope and the number of cells at the time of observation. The expected size of a cleavage-stage human embryo is two equal cells on approximately Day One after fertilization, four cells of the same size on Day Two, until the blastocyst on Day Five. In this study, the embryos of the first group, which were transfected with the EGFP-LINE-1 construct, consisted of cells that were unequal in size and characterized by an increased number of blastomeres in comparison to the cleavage stage of normal embryos. Furthermore, the majority of embryos were characterized by high rates of fragmentation throughout embryo development to cleavage arrest and degeneration at different cleavage stages, mostly before the morula stage. The embryos in the second group, which were transfected with the control vector without the LINE-1 insert, cleaved and developed as normal human embryos (Figure 1 and Figure 2).

**Figure 3:**
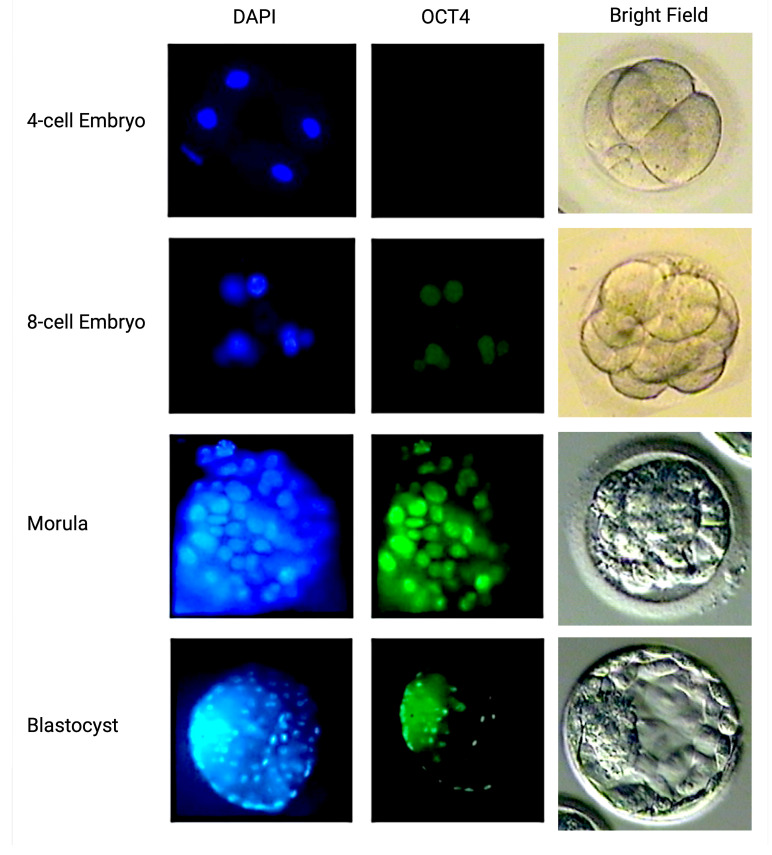
OCT4 protein expression pattern in normal human preimplantation embryos. Oocytes and embryos were double labeled with DAPI for DNA (blue, **left images**) and OCT4 (green, **right images**). Evidence of OCT4 expression was first present in blastomeres of Day 3 embryos.

**Figure 4:**
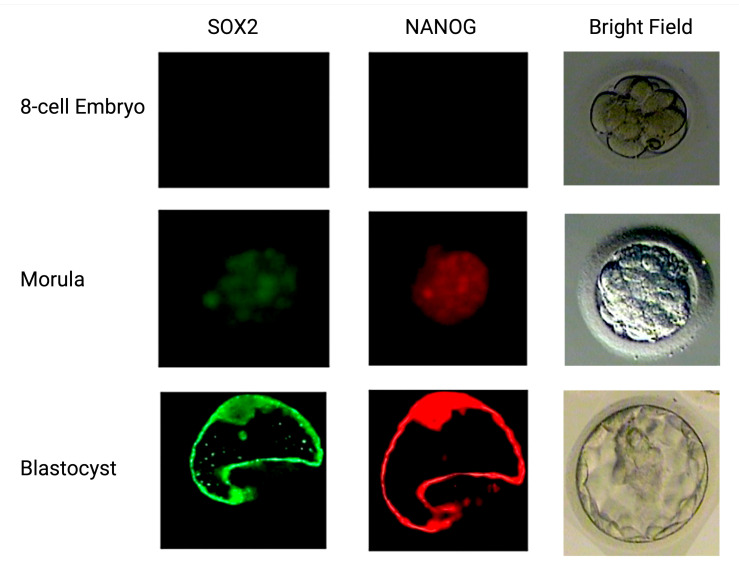
SOX2 and NANOG protein expression in normal human preimplantation embryos. Embryos were stained with FITC secondary antibody for SOX2 (green, **left column**) and Cy3 secondary antibody for NANOG (red, **middle column**) and images were captured by Olympus fluorescence microscope and by Leica Confocal laser microscope for blastocyst stage embryos. SOX2 and NANOG were present from the morula stage, following OCT4 expression on Day 3.

The expression of pluripotency factors during the preimplantation developmental stages presented significant differences between the two embryo groups. In the control group, OCT4 expression was detected for the first time in Day 3 embryos and through the blastocyst stage, where the expression was limited to the inner cell mass (Figure 3). In the LINE-1 injected group, OCT4 presented a similar expression pattern in the early stages of the preimplantation development; it was not further expressed in the morula and blastocyst stages of LINE-1 injected embryos due to cell death. On the other hand, although SOX2 and NANOG were initially expressed at the morula stage of the control embryo group, such expression was not observed at the morula stage of the LINE-1 injected embryos due to their high fragmentation rate and their cleavage arrest (Figure 4).

**Figure 5:**
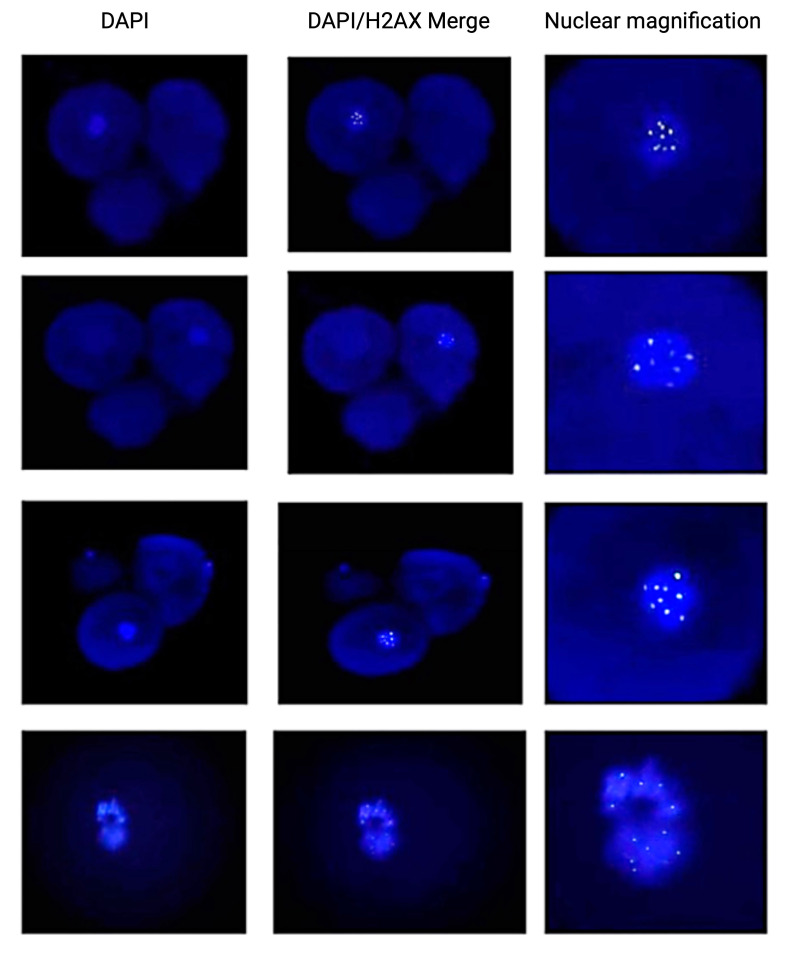
DAPI and H2AX staining of LINE-1 injected embryos with the presence of double-strand DNA breaks. The **left column** depicts the DAPI staining (blue) of the nuclei. The **middle column** shows the merge image of the DAPI and H2AX stains and the **right column** shows nuclear magnifications of selected blastomeres with double-strand DNA breaks.

**Figure 6:**
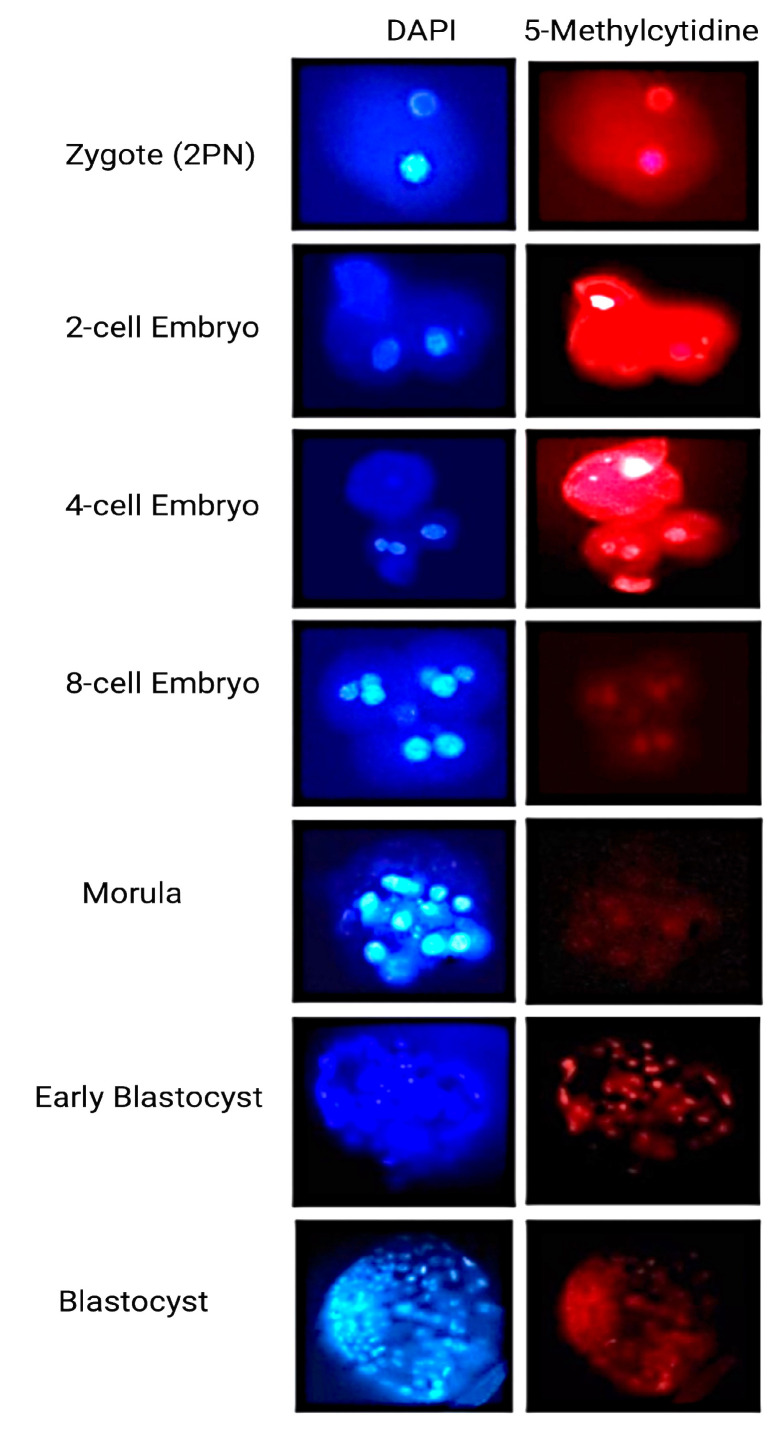
Methylation reprogramming during normal human preimplantation embryo development. Embryos were stained for DNA with DAPI (blue, **left column**) and Cy3 secondary antibody for 5-MeC (red, **right column**) and images were captured by Olympus fluorescence microscope. Methylation was primarily reinstated in the blastocyst stages in the ICM cells.

The blastomeres of the first group of embryos, which had been transfected with the EGFP-LINE-1 construct, were characterized by increased DNA DSBs (Figure 5). However, such a phenomenon was not observed in the control embryos of the second group. Finally, establishment of methylation was observed in the late stages of preimplantation development in control embryos. Methylation patterns in Day one embryos to 16-cell embryos did not differ between the two groups. Concerning methylation patterns at the morula stage and blastocyst stage of the LINE-1-injected embryos, the data were inconclusive due to the high fragmentation rate and the degeneration of the embryos (Figure 6).

## 3. Discussion

Preimplantation human embryos follow a distinct, timely, organized developmental pattern with well-recognized morphological transformations during the five days of in vitro growth to blastocyst. These morphological changes are accompanied by biochemical changes with genetic or epigenetic backgrounds. Various studies have suggested that active expression of retrotransponsable elements in undifferentiated human ESCs during early embryo development affects the genetic and epigenetic mechanisms of mammalian cells, causing genomic instability, gene silencing, and chromosome rearrangements [22,24,25,26,27,28,29,30]. Therefore, it is of great interest to clarify whether certain retrotransposition events are responsible for the morphological changes observed during preimplantation development [31,32]. Even though it is difficult to track these alterations in a living cell, the molecular changes that occur over time may be monitored in preparations of embryos with the appropriate morphological criteria, representing normal developmental stages during preimplantation.

LINE-1 RNAs are carried over from the germ cells into the embryo, and integration occurs during embryogenesis [33,34,35]. To investigate the potential effects of endogenous retrotransposition events on fertilization and early embryo growth, we introduced active LINE-1 retroelements into spare human oocytes matured from MI to MII using ICSI. In the developing embryos, we studied the expression of the most important pluripotency factors for preimplantation development, namely, OCT4, SOX2, and NANOG. The pluripotency factors were analyzed together with the methylation changes that occurred in the early stages of preimplantation development. The monitoring of methylation changes in relation to the expression of pluripotency factors is crucial for understanding the contribution of each pluripotency factor to methylation [36]. We further studied the genome integrity and more specifically, the formation of DNA DSBs in cleavage-stage embryos in response to aberrant expression of LINE-1.

In the control embryo group, OCT4 expression first appeared at the 6–8-cell embryo stage, coinciding with the well-established demethylation, with substitution of methyl groups by hydroxymethyl groups on cytosines, followed by base excision repair. This finding was in accordance with previous studies of OCT4 expression using PCR and immunohistochemistry [37]. SOX2 and NANOG were initially expressed at the morula stage at the time of the second wave of methylation re-establishment, in agreement with documentation in the literature. After blastocyst formation, SOX2 and NANOG retained their expression at high levels, as judged by signal intensity. In blastocysts, OCT4 was mostly expressed in the ICM, whereas SOX2 and NANOG were expressed in both the ICM and the trophectoderm. On the other hand, embryos transfected with exogenous retroelements through ICSI were characterized by remarkable cleavage-stage arrest and the inability to form morula. This was also evident in the immunohistochemistry analysis of pluripotency factors. Even though OCT4 presented with earlier expression (4–6-cell embryo), it was not further expressed in the morula and blastocyst stages due to the high fragmentation of embryos and cell death in the LINE-1-injected embryos. Absence of SOX2 and NANOG expression was also observed in the LINE-1-injected embryos. Retrotransposons are thought to play crucial role early in development and in ESCs by acting as promoters and/or enhancers of key developmental genes such as the three major pluripotency factors, OCT4, NANOG, and SOX2, indicating their potential role in the pluripotent state [38,39,40,41]. The absence of SOX2 and NANOG expression may be secondary to the elevated levels of DNA DSBs due to induced retrotransposition, whereas an abundance of retrotransposons may have an impact on gene expression through insertion into coding regions, premature cleavage of transcripts at poly (A) signals in LINE-1, and genomic rearrangements affecting blastomere growth and blastomere viability, leading to cleavage arrest [42].

Retrotrasposable elements are known from previous reports to produce DNA breaks [22,43,44] as they create new integration sites in the genome, autonomously or non-autonomously, using the appropriate enzymes produced by other intermediating elements. Regarding DNA DSBs, the immunohistochemistry analysis of embryos infected with exogenous LINE-1 showed that they arose as early as the 4-cell stage. Their appearance at this early stage may have been related to the abundance of retrotransposon RNA transcripts found in oocytes making possible the initiation of their replication and integration through exonucleases [27,33,45,46]. Furthermore, the appearance of DNA DSBs at this stage probably had a detrimental effect on the third day of cleavage-stage development that ultimately led to the destruction of the embryo before the morula stage, as observed in this study [47,48]. Recent data demonstrate that levels of the LINE-1 ORF1 protein act as a marker governing oocyte fate. Apoptosis is triggered in oocytes with high levels of LINE-1 ORF1 protein, in order to ensure that LINE-1 retrotransposition during epigenetic reprogramming remains as low as possible in the oocytes and preimplantation embryo [38,49]. Furthermore, regarding LINE-1 ORF2 activity, DNA DSBs generated by the expression of LINE-1 or only by LINE-1 ORF2 can decrease the rate of cells’ entry into mitosis and thereby affect cell survival [43,50]. Similar significantly elevated rates of embryo arrest and decreased clinical pregnancy rates have been observed in couples undergoing ICSI with high levels of nuclear chromatin condensation abnormalities in sperm [51]. Embryo cleavage arrest at the 6- to 8-cell stage, coinciding with the full activation of the embryonic genome, was probably due to increased DNA fragmentation [52] or the inappropriate contribution of the sperm genome and aberrant cell cycle control checkpoints, possibly due to OCT4 expression [27,47,53,54,55,56]. The critical balance between cell cycle control regulators ATM, p53, RB, and others and the effects on early OCT4 expression may be affected by the early-cleavage-stage appearance of DNA DSBs either from the fragmented sperm or by LINE-1 retrotransposition [44,48]. The increased DNA DSBs of the transfected embryos may affect the chromatin structure, which compromises the access of transcriptional factors to the regulatory regions of the genes; thus, the conservation and maintenance of pluripotency is compromised. Consequently, the presence of DNA DSBs at the preimplantation stage of human embryo development, which may be induced by LINE-1 retrotransposition, may harm the embryo’s further development and destabilize DNA integrity, resulting in obstruction of pluripotency.

There are potential limitations to this research, including the restricted number of samples as well as the high fragmentation rate, cleavage arrest, and degeneration of LINE-1 embryos, all of which affected the number of experiments that could be completed. Another restriction is that immunochemistry is less sensitive than other molecular techniques, although it can identify protein expression and localization at the single-cell level. A future step of the current methodology will be the application of the latest, more sensitive technologies, such as RNA sequencing and microarray expression analysis. Ultimately, further research is necessary to verify the results of our study and acquire additional data.

## 4. Materials and Methods

### 4.1. Ethics Approval

The study protocol was designed in accordance with the Helsinki declaration and approved by the Ioannina University Hospital Ethics Committee with protocol number 560 and acceptance date 20 December 2005. Since the study utilized human gametes for research purposes only, the study further received the approval of the Hellenic National Authority of Medically Assisted Reproduction with protocol code 3/2009 and acceptance date 6 July 2009. This embryo research was preceded by animal research in which was shown that human LINE-1 led to abnormal mouse embryo development [57]. This research structure is appropriate according to the ESHRE task force on ethics and law guidelines [58,59,60].

### 4.2. Study Design

In total, 86 immature MI oocytes were donated by twenty infertile couples to the Assisted Reproduction Unit of the Ioannina University Hospital for this study. All couples provided signed informed consent for donation of human gametes for research purposes, and the oocytes were kept in the ART Unit of the Ioannina University Hospital. The donation was strictly for research purposes and any utilization leading to reproduction is prohibited by the local legislation.

**Figure 7:**
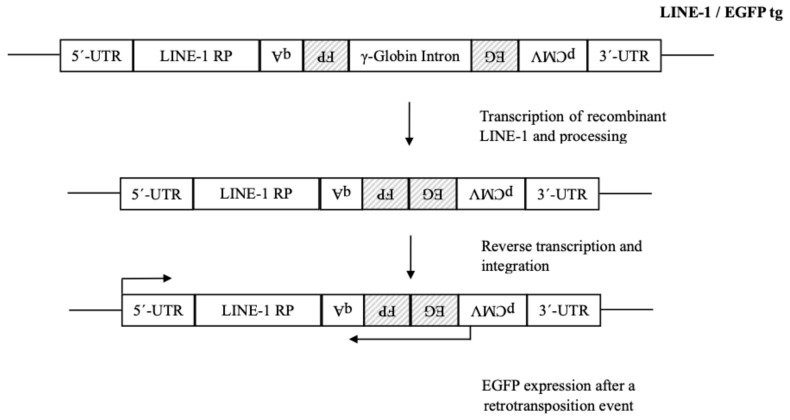
Schematic presentation of human LINE-1 tagged with EGFP retrotransposition cassette. The EGFP retrotransposition cassette is cloned in the 3′-UTR of LINE-1, in antisense orientation. The *EGFP* gene is interrupted by a γ-Globin intron. The *EGFP* gene is expressed only after the transcription, splicing reverse transcription and integration of cDNA into a new chromosomal position, indicating a retrotransposition event. The arrows at the 5′-UTR and the pCMV indicate the transcription orientation of LINE-1 and the *EGFP* gene, respectively.

The in vitro maturation of the above oocytes led to 75 MII oocytes, which were stratified in two groups. Both groups were fertilized with donor sperm acquired from a licensed sperm bank with the appropriate normal parameters. The first group was injected with an EGFP vector carrying a cloned active LINE-1 retroelement (Figure 7) to screen for potential new retrotransposition events, while the second (control group) was injected with the same EGFP vector without the LINE-1 insert. The first and the second group consisted of 52 and 23 embryos, respectively.

### 4.3. Retrotransposition Assay

The presence of de novo retrotransposition events in the transfected embryos was studied using an EGFP-tagged retrotransposition cassette, in which the studied retrotransposons contained the antisense EGFP marker cloned to the 3′-UTR. The *EGFP* gene was transcriptionally controlled by a CMV immediate early promoter, while the presence of a γ-globin intron inserted in the opposite orientation to the *EGFP* gene interrupted the production of functional EGFP protein from the EGFP transcripts. EGFP expression can only be detected when recombinant retrotransposon transcripts undergo splicing, reverse transcription, and integration into the chromosomal DNA, mirroring a retrotransposition event [33,35]. Human oocytes were microinjected with 1–2 pg of plasmid DNA, containing the EGFP-tagged retrotransposons pLINE-1_PR_-EGFP for human LINE-1 [61,62]. Following injection, treated oocytes and early preimplantation embryos were fixed [11] and retrotransposition events were examined via EGFP expression using fluorescence microscopy (Figure 7).

### 4.4. Immunofluorescence Staining and Digital Imaging Microscopy

The removal of zona pellucida was performed chemically using acidified Tyrode’s solution (pH 2.5 ± 0.3). Oocytes and early preimplantation embryos were washed several times in PBS and fixed for 20 min in 4% paraformaldehyde (Paraformaldehyde, extra pure, 500 g, Scharlau PA00950500, Barcelona, Spain). After several washes in PBS, permeabilization occurred with 0.2% Triton X-100 (Triton X, SIGMA-Aldrich T8787, St. Louis, MO, USA) in PBS for 15 min at room temperature. For the detection of 5-methyl-cytosine (5-MeC), zygotes were treated with HCL 4Ν (Hydrochloric acid 320331, Sigma-Aldrich, ACS reagent, 37%) at room temperature for 10 min to remove purines and denatured DNA. Subsequently, the samples were neutralized for 10 min with 100 mM Tris/HCl buffer, pH 8, after permeabilization. To eliminate non-specific fluorescence, the samples were blocked overnight at 4 °C in 1% BSA, 0.05% Tween 20 in PBS (PBST) (Tween-20, Sigma-Aldrich P1379). The samples were incubated for 1 h at room temperature in the dark with specific primary antibodies against OCT4, SOX2 NANOG, γH2AΧ, or 5-methyl-cytosine [Oct-3/4 antibody Mouse monoclonal IgG2b (C-10) (Ref: sc-5279, Santa Cruz, Dallas, TX, USA), Anti-SOX2 antibody Mouse monoclonal [9-9-3] (Ref: ab79351, ABCAM, Cambridge, UK), Anti-Nanog antibody Rabbit polyclonal to Nanog (Ref: ab80892, ABCAM), Anti-gamma H2A.X (phosphor S139) Rabbit IgG polyclonal antibody (Ref: ab11174, ABCAM), Anti-5-Methylcytidine monoclonal antibody vs. 5-Methylcytidine, (Mouse IgG1/λ, Purified Ascites,100 μg, Ref: BI-MECY-0100, Eurogentec, Liège, Belgium). After extensive washing with 0.05% Tween 20 in PBS, samples were incubated for 1 h at room temperature in the dark with a secondary antibody specific against each primary antibody, coupled with fluorescein [Alexa Fluor 488-conjucated AffiniPure Donkey Anti-Rabbit IgG (H + L) (Jackson Immunoresearch, West Grove, PA, USA, Ref: 711-545-152), Cy3-conjucated AffiniPure Donkey Anti-Mouse IgG (H + L) (Jackson Immunoresearch, Ref: 715-165-150), Cy3 Donkey Anti-Rabbit IgG (H + L) (Jackson Immunoresearch, Ref: 711-165-152), Fitc Donkey Anti-Mouse IgG (H + L) (Jackson Immunoresearch, Ref: 715-095-150), Cy3 Donkey Anti-Rat IgG (H + L) (Jackson Immunoresearch, Ref: 712-165-150). DNA was stained with 3 mg/mL 4,6-diamidino-2- phenylindole (DAPI) and samples were mounted in antifade solution (ProLong^®^ Gold Antifade Reagent, Invitrogen, Waltham, MA, USA, Ref Num P36930). An Olympus BX40 fluorescence microscope and a Leica TCS SP5 confocal laser scanning microscope with LASAF software (version 2.4) were used to detect fluorescence in the above analyses.

## 5. Conclusions

In conclusion, our findings highlight the expression patterns of the pluripotency factors OCT4, SOX2, and NANOG in respect to methylation and LINE-1 retroelement experimental activation during the preimplantation developmental stages. Our study also demonstrates that the very early initiation of DNA DSBs because of the LINE-1 experimental activation interfered with further preimplantation embryo development to the blastocyst stage and hindered the positive influences of OCT4, SOX2, and NANOG on the formation of the morula, the ICM, and the trophectoderm.

## Data Availability

The raw data supporting the conclusions of this article will be made available by the corresponding author on request.
